# The amphibian peptide Yodha is virucidal for Zika and dengue viruses

**DOI:** 10.1038/s41598-020-80596-4

**Published:** 2021-01-12

**Authors:** Song Hee Lee, Eui Ho Kim, Justin T. O’neal, Gordon Dale, David J. Holthausen, James R. Bowen, Kendra M. Quicke, Ioanna Skountzou, Shyla Gopal, Sanil George, Jens Wrammert, Mehul S. Suthar, Joshy Jacob

**Affiliations:** 1 Emory Vaccine Center, Yerkes National Primate Center, Emory University, 954 Gatewood Road, Atlanta, GA 30329 USA; 2 Viral Immunology Laboratory, Institut Pasteur Korea, Seongnam, Republic of Korea; 3 Division of Infectious Diseases, Department of Pediatrics, Emory University School of Medicine, Atlanta, GA 30322 USA; 4 Rajiv Gandhi Center for Biotechnology, Poojapura, Thiruvananthapuram, Kerala 695014 India

**Keywords:** Virology, Dengue virus, Pathogens

## Abstract

Zika virus (ZIKV) has emerged as a serious health threat in the Americas and the Caribbean. ZIKV is transmitted by the bite of an infected mosquito, sexual contact, and blood transfusion. ZIKV can also be transmitted to the developing fetus in utero, in some cases resulting in spontaneous abortion, fetal brain abnormalities, and microcephaly. In adults, ZIKV infection has been correlated with Guillain–Barre syndrome. Despite the public health threat posed by ZIKV, neither a vaccine nor antiviral drugs for use in humans are currently available. We have identified an amphibian host defense peptide, Yodha, which has potent virucidal activity against ZIKV. It acts directly on the virus and destroys Zika virus particles within 5 min of exposure. The Yodha peptide was effective against the Asian, African, and South American Zika virus strains and has the potential to be developed as an antiviral therapeutic in the fight against Zika virus. The peptide was also effective against all four dengue virus serotypes. Thus, Yodha peptide could potentially be developed as a pan-therapeutic for Zika and dengue viruses.

## Introduction

Zika virus (ZIKV) is a mosquito-borne virus that belongs to the family *Flaviviridae*. It is closely related to the West Nile virus (WNV), dengue virus (DENV), and Japanese Encephalitis virus (JEV)^1^. Similar to DENV, ZIKV is spread through the bite of an infected *Aedes sp*. mosquito^2,3^. Unlike other flaviviruses, ZIKV can be vertically transmitted from an infected mother to the developing fetus in *utero*, resulting in congenital Zika syndrome, which is characterized by spontaneous abortion, fetal brain abnormalities, and microcephaly^4–8^. In adults, ZIKV has also been linked to Guillain–Barre syndrome, a disorder in which the immune system attacks the nervous system. ZIKV can be divided into four distinct lineages based on sequence homology^9,10^. The first ZIKV, MR-766, was isolated in Uganda from a sentinel rhesus monkey in 1947. In 1966, the first non-African strain P6-740 was isolated in Malaysia from a pool of *Aedes aegypti* mosquitoes. The African strain, DaKar41524, was isolated from a pool of *Aedes africanus* mosquitoes from Senegal in 1984. In 2015, the contemporary strain, PRVABC59, was isolated in Puerto Rico from an infected human patient. This strain, which is closely related to the epidemic strains circulating in the Americas have been linked to in utero ZIKV infection. Recently, it has been shown that the Brazilian strain of ZIKV causes congenital disabilities in an experimental mouse^11–13^. Employing this mouse model, a vaccine has been developed that offers complete protection in susceptible mice against ZIKV challenge^14,15^. However, there are significant concerns regarding the efficacy of this vaccine in flavivirus-experienced individuals^16,17^. A potential strategy to combat ZIKV infection would be to develop antiviral therapeutics to treat or prevent disease.

Host defense peptides constitute an ancient arm of the innate immune system. They comprise a diverse class of naturally-produced peptides that serve as the first line of defense in all living, uni- and multi-cellular organisms^18–22^. They neutralize pathogens by either killing them directly by physically disrupting the outer membrane or by blocking internal functions^23^. We isolated host defense peptides from the skin of the frogs found in the Western Ghats of southwestern India^24^. Recently, we discovered a peptide that has potent antiviral activity against influenza virus^25^. We hypothesized that although these peptides have been selected to protect the amphibian against pathogens in their habitat, some of these peptides could also cross neutralize ZIKV and dengue viruses.

## Results

### A novel peptide from Indosylvirana aurantiaca exhibits anti-Zika virus activity in vitro

**Figure 1:**
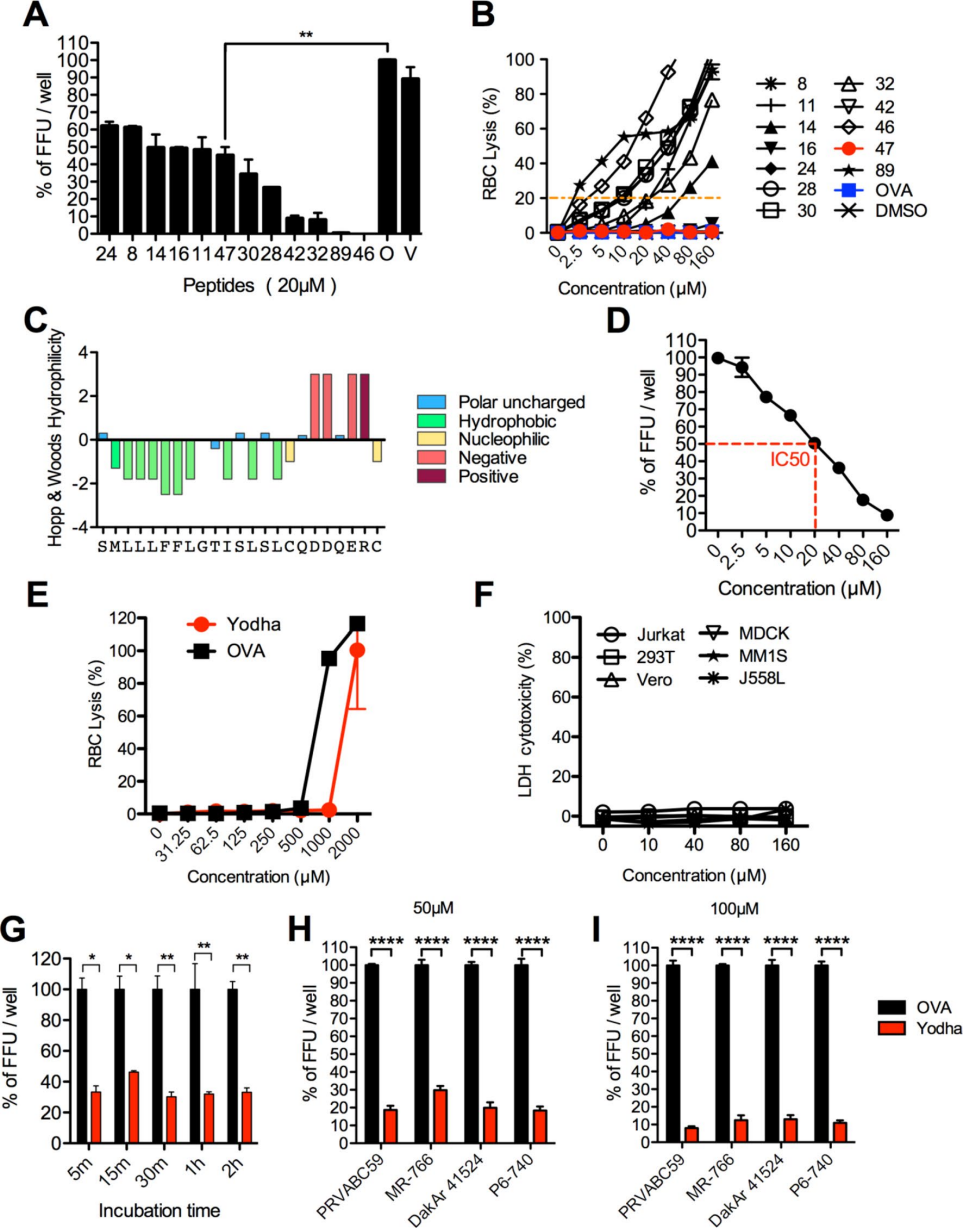
Screening and identification of frog peptides against Zika virus (**A**) Host defense peptides were incubated with ZIKV for two hours and virus viability was tested using focus-forming assay (FFA) of ZIKV at 72 h post infection. OVA peptide used as a negative control (set as an 100% of FFU/well). *T-test*, Two-tailed p value p = 0.0077 (**). (**B**) Cytotoxicity of each peptide was tested using hemolysis. Briefly, 2 × 10^7^ Human red blood cells were treated with increasing concentrations of each peptide and RBC lysis. (**C**) Amino acid sequence of Yodha is represented on the Hopps & Woods amino acid hydrophilicity scale. (**D**) IC_50_ measurement of Yodha in ZIKV infection by FFA. Data are means ± SEM from at least three independent experiments. (**E**) Cytotoxicity of Yodha and OVA peptide were tested using higher concentrations of up to 2000uM against human RBCs. Human red blood cells were treated with increasing concentrations of each peptide and RBC lysis was mesaured. (**F**) LDH Cytotoxicity of Yodha peptide was tested using higher concentrations of up to 160uM by measuring LDH release in various cells(Jurkat, MM1S, 293 T, MDCK, Vero and J558L). (**G**) Kinetics of Yodha induced ZIKV inhibition was examined over time (5 min to 2 h) (peptide concentration 20 µM). OVA peptide was used as a positive control (set as an 100% of FFU/well). Values represent mean ± S.E.M. *P < 0.05 and **P < 0.01; by Two-way ANOVA. Zika virus strains PRVABC59, MR-766, DakAr 41,524, and P6-740 were exposed to Yodha or control OVA peptide at (**H**) 50 µM (**I**) 100 µM concentration. Values represent mean ± S.E.M. ****p < 0.0001; by Two-way ANOVA.

We screened frog skin peptides to identify peptides with potentially virucidal activity against ZIKV infection. Briefly, we incubated individual peptides with ZIKV (PRVABC59 strain) for 2 h and then tested viral viability in a focus-forming assay. Of the library of 76 peptides, 12 peptides decreased ZIKV infectivity (Fig. 1A). A significant drawback of host defense peptides is that they can be toxic to mammalian cells. To identify non-toxic candidates among the 12 peptides, we measured the toxicity of each peptide against human erythrocytes. Only one of the 12, peptide 47, showed no cytotoxicity even at high concentrations (Fig. 1B). We named this peptide, Yodha, which in Sanskrit means ‘warrior.' The Yodha peptide contains 23 amino acids (SMLLLFFLGTISLSLCQDDQERC) and belongs to the Brevenin superfamily. We analyzed this peptide using the Hopp and Woods plot^26^, and amino acids with hydrophobic side chains dominate almost half of the N-terminal region of Yodha peptide (Fig. 1C). The C-terminus of Yodha peptide contains three negatively charged amino acids.

To determine the half-maximal inhibitory concentration (IC_50_), we performed a dose-escalation analysis of the Yodha peptide (2.5 µM to 160 µM) against ZIKV and observed 35%, 50%, 65%, 80% and 90% reductions at 10 µM, 20 µM, 40 µM, 80 µM, and 160 µM, respectively. The IC_50_ of Yodha peptide is 20 µM (Fig. 1D). To determine the maximum concentration of Yodha that could be used without toxicity, we redid the human red blood cell (RBC) cytotoxicity test at peptide concentrations ranging from 31.25 µM to 2000 µM. Yodha was nontoxic even at 1000 µM and showed toxicity only at 2000 µM (Fig. 1E). In addition to RBC lysis-cytotoxicity assay, we performed an LDH cytotoxicity test to observe cell membrane damage and necrotic cell death in various cells such as human cells (Jurkat, MM1S, and 293 T), mouse cells (J558L), Monkey cells (Vero), and canine cells (MDCK). Yodha was nontoxic at 160 µM in all cell lines tested (Fig. 1F).

Next, we sought to determine the kinetics of viral reduction by this peptide following exposure to ZIKV. We exposed ZIKV to Yodha peptide for 5 min, 15 min, 30 min, 1 h, and 2 h. Viral titers (measured as Focus Forming Unit, FFU) were significantly decreased at all time points tested and strongly indicates that the maximum activity of the Yodha peptide occurs within the first five minutes of incubation with ZIKV (Fig. 1G).

For all our experiments in Fig. 1, we used the PRVABC59 (Puerto Rico 2015) strain of ZIKV. Hence, next we sought to determine the extent to which Yodha peptide would inhibit the different strains of ZIKV, MR-766 (Uganda, 1947), DakAr41524 (Senegal 1984), P6-740 (Malaysia, 1966) and PRVABC59 (Puerto Rico, 2015). The Yodha peptide inhibited all ZIKV strains (Fig. 1H,I). Taken together, our data suggest that Yodha peptide might target a common region that is shared by these four ZIKV strains.

### Yodha peptide can reduce viral titers and limit the spread of ZIKV in vitro

**Figure 2:**
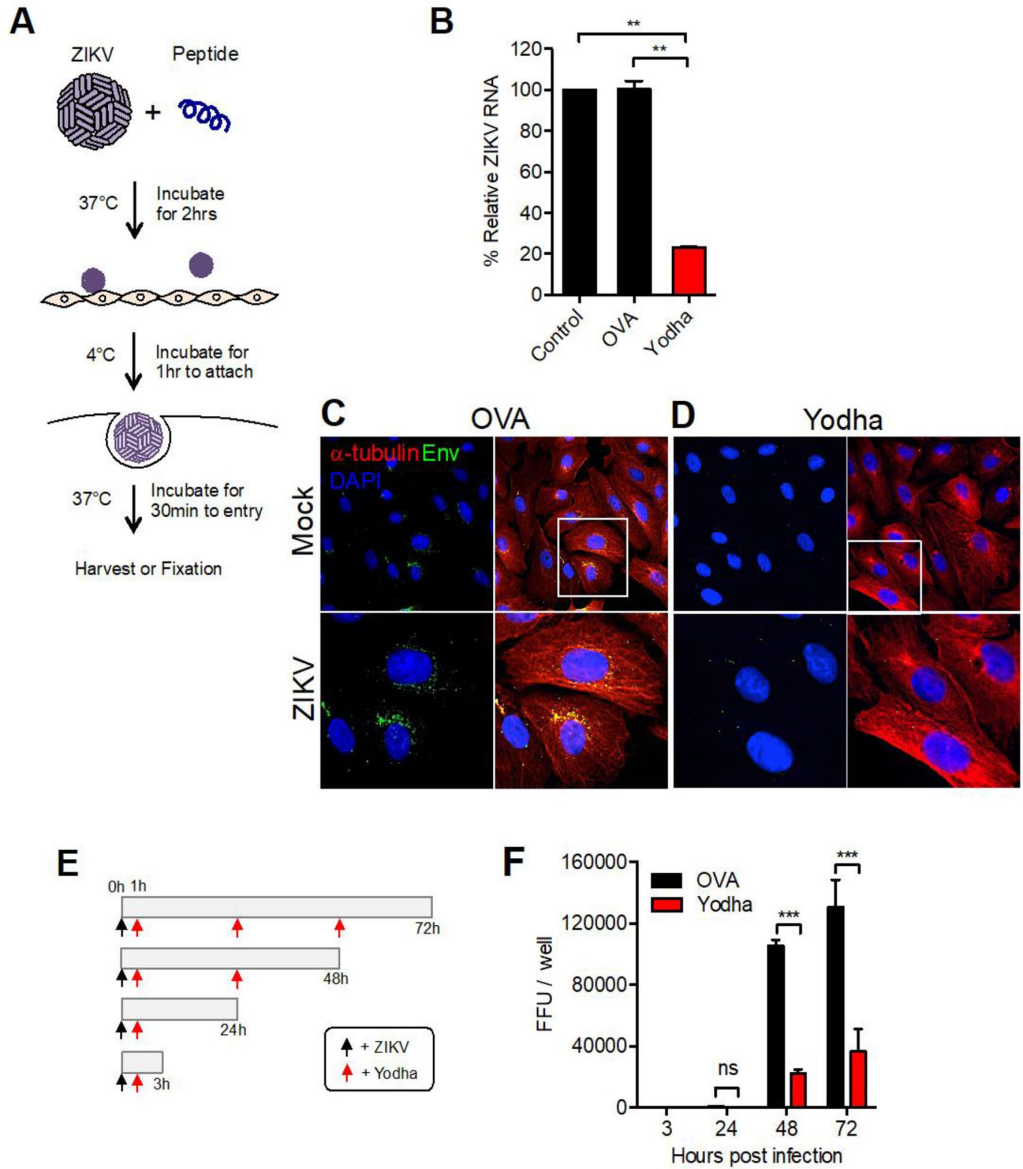
Yodha peptide inhibits ZIKV entry into cells. (**A**) Schematic diagram of the experimental setup. (**B**) ZIKV was treated with 200 µM of Yodha or control OVA peptide for 30 min and then used to infect Vero cells at a MOI of 0.5 and expression of ZIKV RNA was determined by quantitative RT-PCR (qRT-PCR). Cellular glyceraldehyde-3-phosphate dehydrogenase (GAPDH) served as internal control. T-test two tailed (**) p = 0.0028. (**C**) Mock-infected and ZIKV-infected Vero cells were fluorescently stained for ZIKV envelope (green), a-tubulin (red) and DAPI (blue). ZIKV was exposed to control ova peptide or Yodha peptide. Images were taken with an Olympus Fluoview FV1000 microscope using FV10-ASW2.1 acquisition software (https://www.olympus-lifescience.com/en/support/downloads/fv10i_vw_license/). (**D**) and then used to infect Vero monolayers. ZIKV envelope was visible inside the cytoplasm in control but not Yodha-peptide treated Vero cells. (**E**) Schematic of the experimental design is shown. Briefly, Vero cell monolayers were infected first with ZIKV and then treated with Yodha on control OVA peptides (40 µM) at 1 h, 24 h, 48 h and 72 h post-infection. Culture supernatants were harvested at 3, 24, 48, and 72 h post infection and assayed for ZIKV. (**F**) Treatment with Yodha peptide led to significantly reduced ZIKV titers in the supernatants. Error bars indicate the SEM of four technical replicates statistical significance was assessed by a 2-way ANOVA.

Next, we determined whether the observed Yodha peptide-induced decrease in viral titers was due to blocked viral entry. We reasoned that if the peptide directly kills or disrupts the envelope structure of ZIKV, this would prevent virus entry. To test this, we designed an experiment to measure virus entry, by allowing ZIKV to bind cells at 4 °C for 1 h followed by shifting the cells to 37 °C to permit virus entry (Fig. 2A). It has been shown that DENV2 entry occurs within 25 min by early endosome trafficking^27^. Briefly, we exposed ZIKV to Yodha peptide and then allowed the virus to infect Vero cells for 30 min. We then measured viral RNA within the Vero cells by quantitative Real Time-PCR (qRT-PCR) (Fig. 2B). Comparable amounts of ZIKV RNA were detected in the virus-only samples and control samples of ZIKV incubated with control OVA peptide. Interestingly, cells infected with ZIKV that were pre-incubated with Yodha peptide had only 20% of the ZIKV RNA expressed compared to the OVA and virus-only controls, suggesting that exposure to Yodha peptide significantly reduced the ability of ZIKV to enter the host cells. Next, we visualized ZIKV entry or lack thereof by immunofluorescence using antibodies against pan flavivirus E protein. As expected from the qRT-PCR results, we observed decreased expression of ZIKV envelope in Yodha peptide-treated samples (Fig. 2D), while the OVA control (Fig. 2C) showed no such effect. This suggests that Yodha peptide inhibits ZIKV and prevents viral entry into cells. Next, we sought to determine if Yodha peptide could reduce infectious virus production in cells already infected with ZIKV. Briefly, we infected Vero cells, allowed virus attachment and entry, and then added Yodha peptide to the culture medium at 1, 24, 48, and 72 h post-infection. We collected culture supernatants at 3, 24, 48, and 72 h after ZIKV infection to quantitate ZIKV by FFU assay (Fig. 2E). ZIKV was not detected at 3 h post-infection in both OVA- or Yodha-treated cells. We observed a 60–70% reduction in ZIKV production at 48 and 72 h post-infection (Fig. 2F). Taken together, the data shows that Yodha peptide can significantly reduce viral titers and limit the spread of ZIKV in vitro.

### Yodha disrupts Zika virus integrity

**Figure 3:**
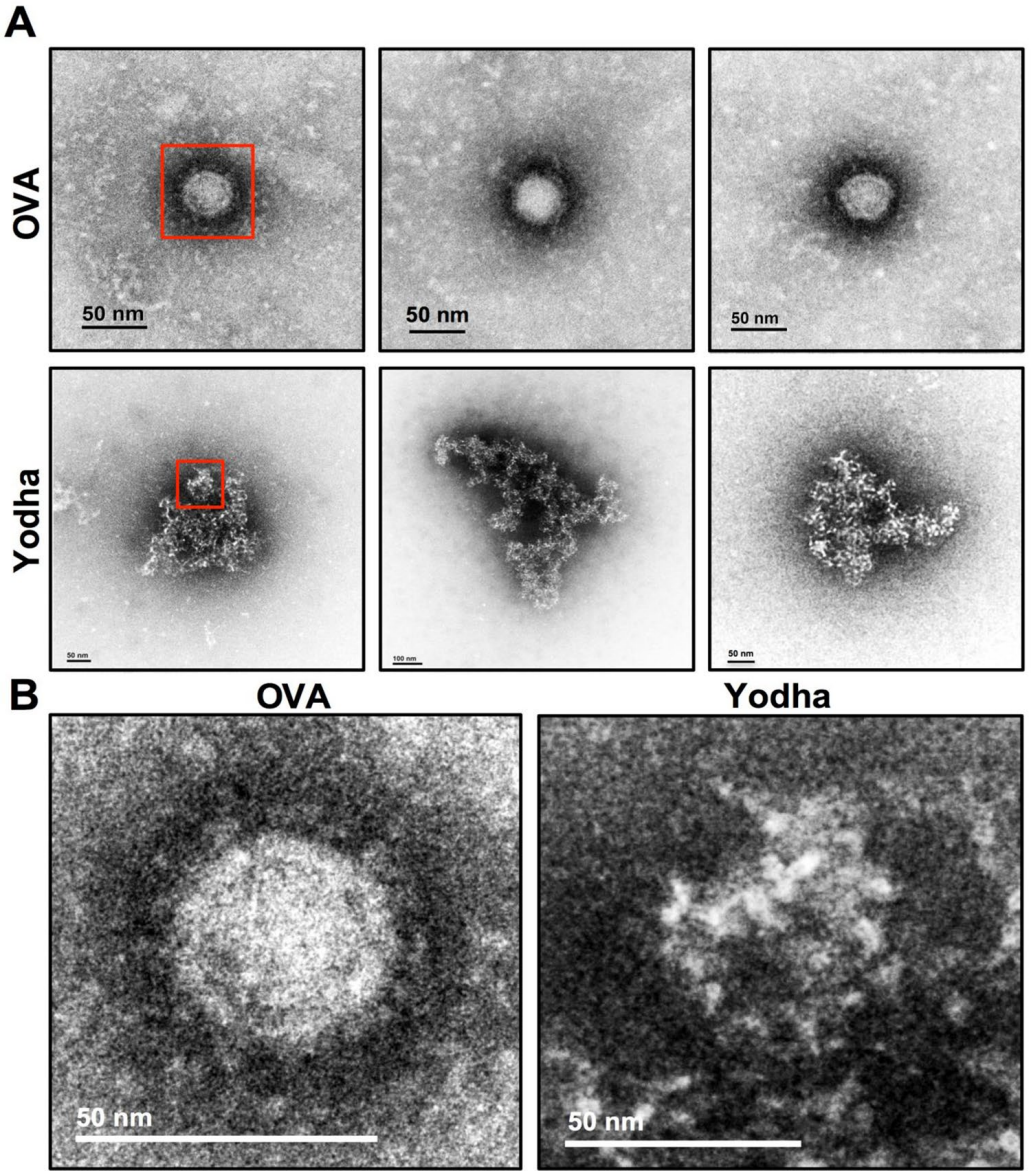
Transmission electron microscopy shows that Yodha peptide destroys ZIKV. (**A**) TEM micrographs of ZIKV treated with OVA (control) or Yodha peptide are shown. ZIKV were incubated with the peptides at 200 µM for just 10 min, fixed with paraformaldehyde and then processed for electron microscopy. Yodha treatment led to loss of morphology and aggregation of virus particles. (**B**) Enlarged images of ZIKV treated with control or Yodha peptide from (**A**) are shown. The data is representative of three experiments performed.

We showed in Fig. 2 that exposure of ZIKV to Yodha peptide inhibited viral entry, and this effect could be due to peptide binding to the virus and blocking entry, or the peptide could be destroying the virus. To test this, we treated ZIKV with Yodha peptide or control peptide for 10 min and analyzed virus particles by electron microscopy. Surprisingly, a 10-min treatment with Yodha peptide-induced disruption of viral particles in contrast to the control peptide treated ZIKV, which exhibited intact virions (Fig. 3). ZIKV treated with Yodha peptide showed significant structural disruption, as seen by the loss of a well-circumscribed morphology, loss of clearly defined layers, and formation of aggregates composed of disrupted viral particles – a feature that has also been observed in defensin-induced viral aggregation^28,29^. Taken together, these results suggest that ZIKV treated with Yodha peptide loses its infectivity due to the physical disruption of the Zika virion.

### Yodha requires sequence fidelity for activity

**Figure 4:**
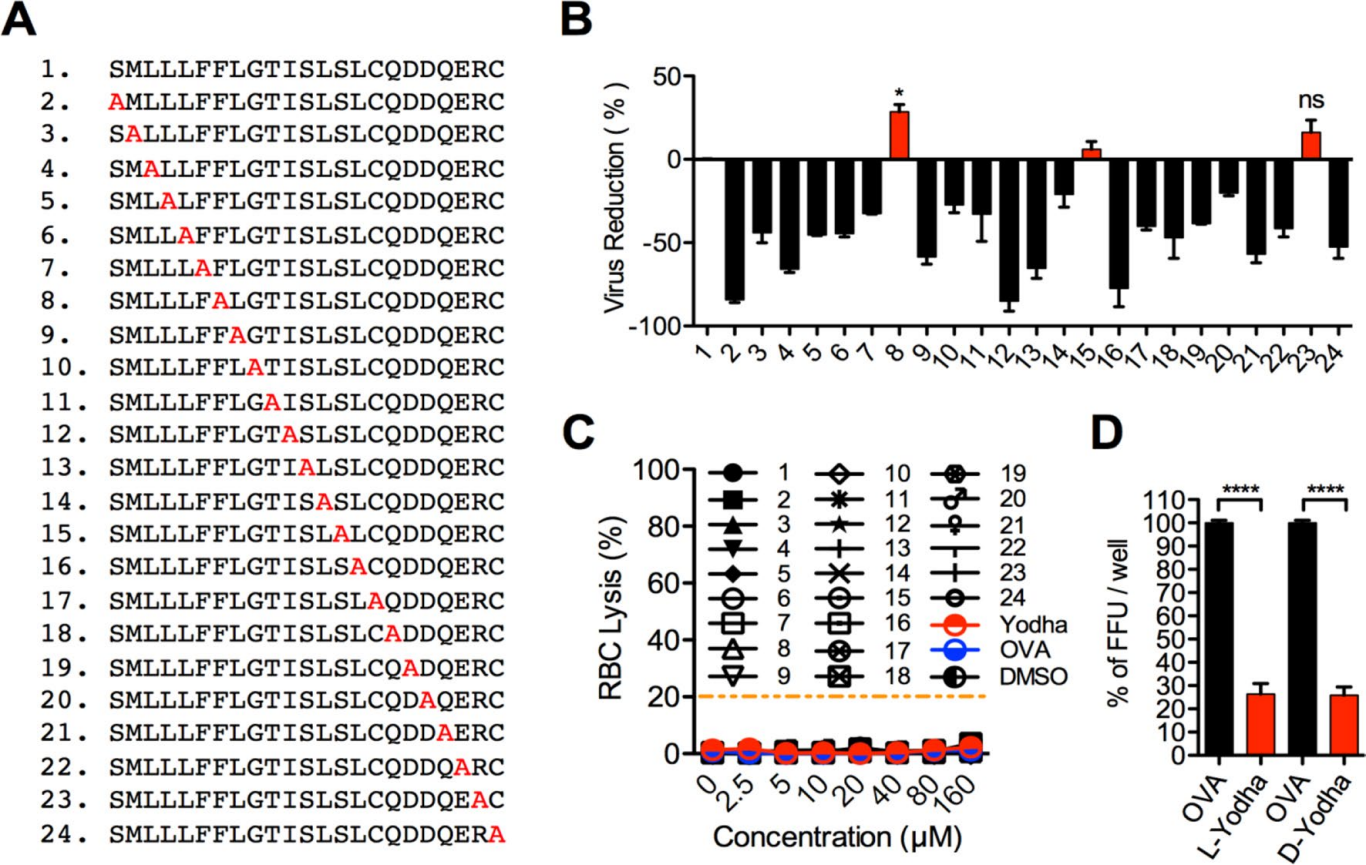
Alanine-scanning mutants of Yodha (**A**) List of alanine-scanning mutants of Yodha. (**B**) Change in virucidal activity of alanine-scanning mutant peptides compared with Yodha peptide which was set as zero. *T-test* two tailed p-value (*) p = 0.021, (ns) Non significant p = 0.1553. (**C**) Each of the alanine scan mutants was tested for cytotoxicity test using human red blood cells. (**D**) Both, the L-enantiomer of Yodha (L-Yodha), and the D-enantiomer (D-Yodha) exhibited comparable anti-viral activity against ZIKV.

To further characterize Yodha peptide, we generated alanine-scanning mutants of the peptide, which removes the side chain, but maintains peptide conformation, allowing for the assessment of the significance of each amino acid within the larger structure (Fig. 4A). Each of 23 mutated peptides was tested for its (a) ability to neutralize ZIKV (Fig. 4B) and (b) toxicity against human RBC, if any (Fig. 4C). Interestingly, only one of the alanine mutants (peptide 8) showed statistically significant (p < 0.02) antiviral activity than the parental peptide, while 20 out of 23 mutants had decreased activity and two mutants exhibited comparable activity as Yodha peptide (Fig. 4B). None of the ALA mutants were toxic and caused lysis of RBCs (Fig. 4C). Chirality is an essential factor to consider for pharmacological application since the mirror-image, D-enantiomer peptide, is more stable in vivo than the naturally occurring L-enantiomer. Interestingly, both D- and L-form of peptide neutralized ZIKV with comparable efficiency (Fig. 4D), suggesting that this peptide might target a symmetric structure in the virus that is recognized by both D and L form of the peptide.

### Naturally occurring variants of Yodha are also effective against ZIKV infection

**Figure 5:**
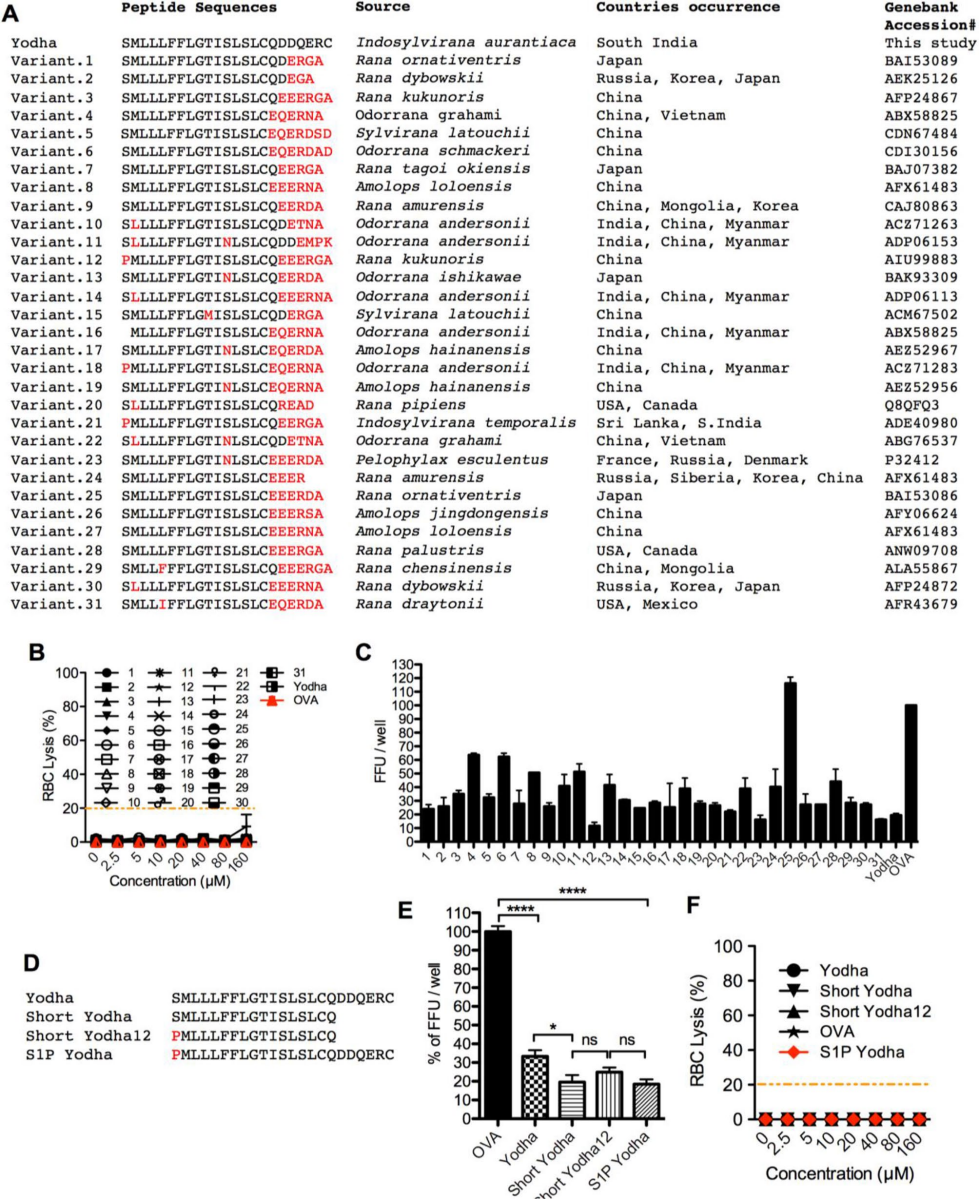
Naturally-occurring variants of Yodha peptides inhibit ZIKV. (**A**) A list of natural variants of the Yodha peptide, the identity and geographical distribution of amphibians that produce these peptides are shown. (**B**) Cytotoxicity tests show that the Yodha peptide variants are non-toxic to human RBCs. (**C**) Each of these naturally occurring variants of Yodha peptide was tested for their ability to neutralize ZIKV. All variants but one inhibited ZIKV. (**D**) Truncated and mutant Yodha peptides tested are shown. The ability of these to inhibit ZIKV is shown in (**E**) and the lack of toxicity of these are shown in (**F**). Statistical significance was assessed by T-test; two tailed. * p = 0.0215, ns; not significant, and ****p < 0.0001.

The *Indosylvirana aurantiaca* produces Yodha peptide almost certainly, not to specifically fight Zika viruses but perhaps to combat other pathogen(s) the amphibian would encounter in its niche. The Yodha peptide appears to be analogous to signal sequences found upstream of host defense peptides. So next, we sought to determine how common this peptide or its analogs are in host defense peptides isolated from elsewhere in the world. We searched using the BLASTp (https://blast.ncbi.nlm.nih.gov/Blast.cgi?PAGE=Proteins) program and identified 31 peptides that exhibited high levels of homology, especially in the hydrophobic N-terminus (Fig. 5A). We then determined the extent to which any of these variant peptides can neutralize ZIKV. First, we tested their toxicity, and none of these variant peptides showed toxicity to human RBCs (Fig. 5B). Interestingly, all of the variant peptides except one showed virucidal activity against ZIKV (Fig. 5C); variant 25, which is identical to Yodha except for the 6 C terminal amino acids exhibited no virucidal effect on the virus. On the other hand, variant #12 (brevinin-2KK2 from [*Rana kukunoris*], PMLLLFFLGTISLSLCQEEERGA) demonstrated 1.6-fold increased effect against ZIKV than Yodha peptide. On the N-terminus, Yodha peptide and variant #12 are identical except for position 1, which is changed from a Serine to a Proline residue; the C terminus of these peptides differ drastically after position 17(Q). Interestingly, variant 21 (PMLLLFFLGTISLSLCQEERGA), which was isolated from the frog, *I. temporalis* hailing from the same geographical area as the Yodha peptide-expressing frog *Indosylvirana aurantiaca,* presented the S1-P change but had one less glutamic acid amino acid at the terminus also exhibited activity against ZIKV. We next investigated whether the S to P change at position one or removing the C-terminus (sequences after Q17) contributes to the improved activity. We also generated a series of mutant Yodha peptides, including one which lacks the C terminal DDQERC, a short 12 peptide fragment that lacks the EEERGA from variant #12, and a S1P peptide that replace S1-P in the full-length Yodha (Fig. 5D) and tested their activity against ZIKV (Fig. 5D). We found that the S1P full-length Yodha peptide was significantly more effective against ZIKV than the WT Yodha peptide. We also saw that removal of the C terminus from Yodha, as well as variant #12, significantly improved activity (Fig. 5E). This also suggests that the activity lies in the N terminal hydrophobic region. All of the truncated Yodha peptides were nontoxic to human RBCs (Fig. 5F). Taken together, these results suggest that either changing S-P at position one at the N terminus or removing the C terminal end—DDQERC improves the activity of the Yodha peptide against Zika viruses.

### Yodha reduces ZIKV viremia and viral load in vivo

**Figure 6:**
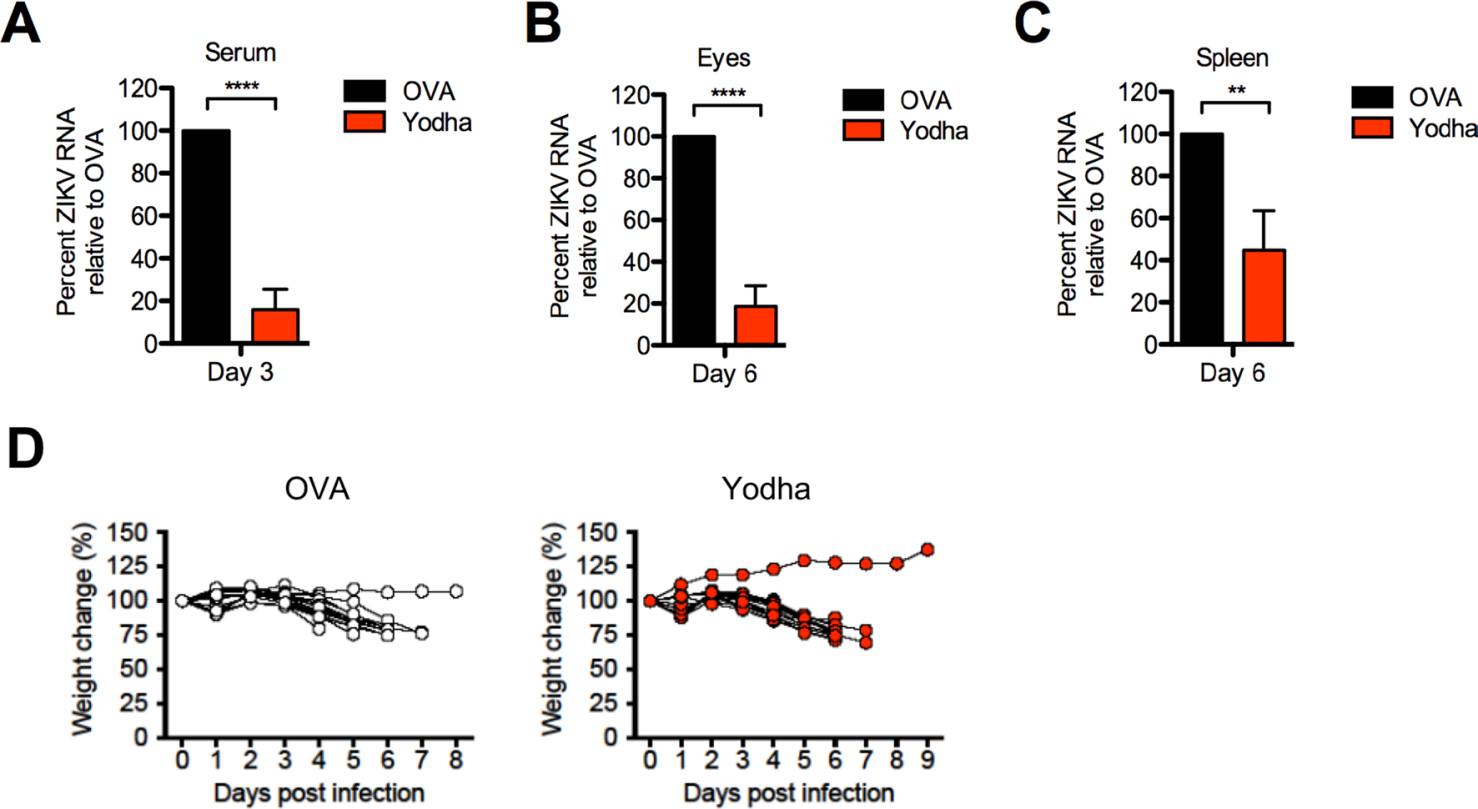
Administration of Yodha peptide reduces ZIKV viremia and viral burden in mice. Briefly, cohorts of 4–5-week-old mice were given 2 mg anti-Ifnar1 mAb intraperitoneally, and the following day infected with 10^5^ FFUs of PRVABC ZIKV. Viremia and viral burden were monitored in serum and tissues by real time PCR. Viremia in the serum at days 3 post-infection (**A**) samples and viral burden at day 6 in the eye (**B**) and spleen (**C**) are shown. All qRT-PCR data is represented as a relative percentage as compared to the average of the OVA controls, calculated as: percent ZIKV RNA relative to OVA = ([Yodha_ZIKV RNA_] / [Average OVA_ZIKV RNA_]) * 100. (**D**) Cohorts of 5–6-week-old IFNAR KO mice were infected with 10^2^ FFUs of ZIKV MR766. Body weights were measured daily and the changes for OVA- and Yodha-treated groups are displayed. Statistical significance was assessed by unpaired *t-*test; two-tailed (**A**, Left) p < 0.0001 **** (**A**, Right) p = 0.0125 *, (**B**) p < 0.0001 **** (**C**) p = 0.0088 **.

We have established that Yodha has anti-ZIKV activity in vitro, and next, we examined if it was functional in vivo and could lower virus titers in mice in a ZIKV infection model. Using unformulated peptides to test efficacy in vivo in animals is sub-optimal; nonetheless, we used it to verify proof of principle that Yodha can function in vivo. For these experiments, we used 4-week-old immunocompetent C57BL/6 mice that were treated with a single i.p injection of 2 mg anti-IFNAR1 monoclonal antibody to impair type I IFN signaling and the following day, injected subcutaneously with PRVABC ZIKV as described^11,30^. Briefly, animals were treated with anti-IFNAR1 antibody, 24 h later treated with Yodha (D-form) or control OVA peptide (i.p; 0.1 mg/mouse), and then infected subcutaneously 5 h later in the footpad with 10^5^ focus forming units of ZIKV strain PRVABC. Mice received Yodha or OVA peptide injections every day for the next 3 days. We measured ZIKV RNA in serum by qRT-PCR on days 3 post-infection (Fig. 6A). Yodha-treated mice showed significantly reduced viremia as compared to OVA-treated mice (Fig. 6A). We also determined the viral RNA load in the eyes (Fig. 6B) and spleen (Fig. 6C) and found significantly reduced viral loads in the Yodha peptide-treated group. Taken together, these data suggest that Yodha efficiently decreased viral load in vivo, consistent with the in vitro results.

Anti-IFNAR 1 antibody-treated immunocompetent C57BL/6 mice, upon infection with PRVABC ZIKV do not exhibit weight loss or death. To assess morbidity, we switched to IFNAR KO mice and infected them with the MR766 ZIKV strain. In this more aggressive virus infection model, we did not observe any differences in survival (data not shown) or weight changes (Fig. 6D) between the Yodha peptide-treated and control groups.

### Yodha is effective against all dengue virus serotypes

**Figure 7:**
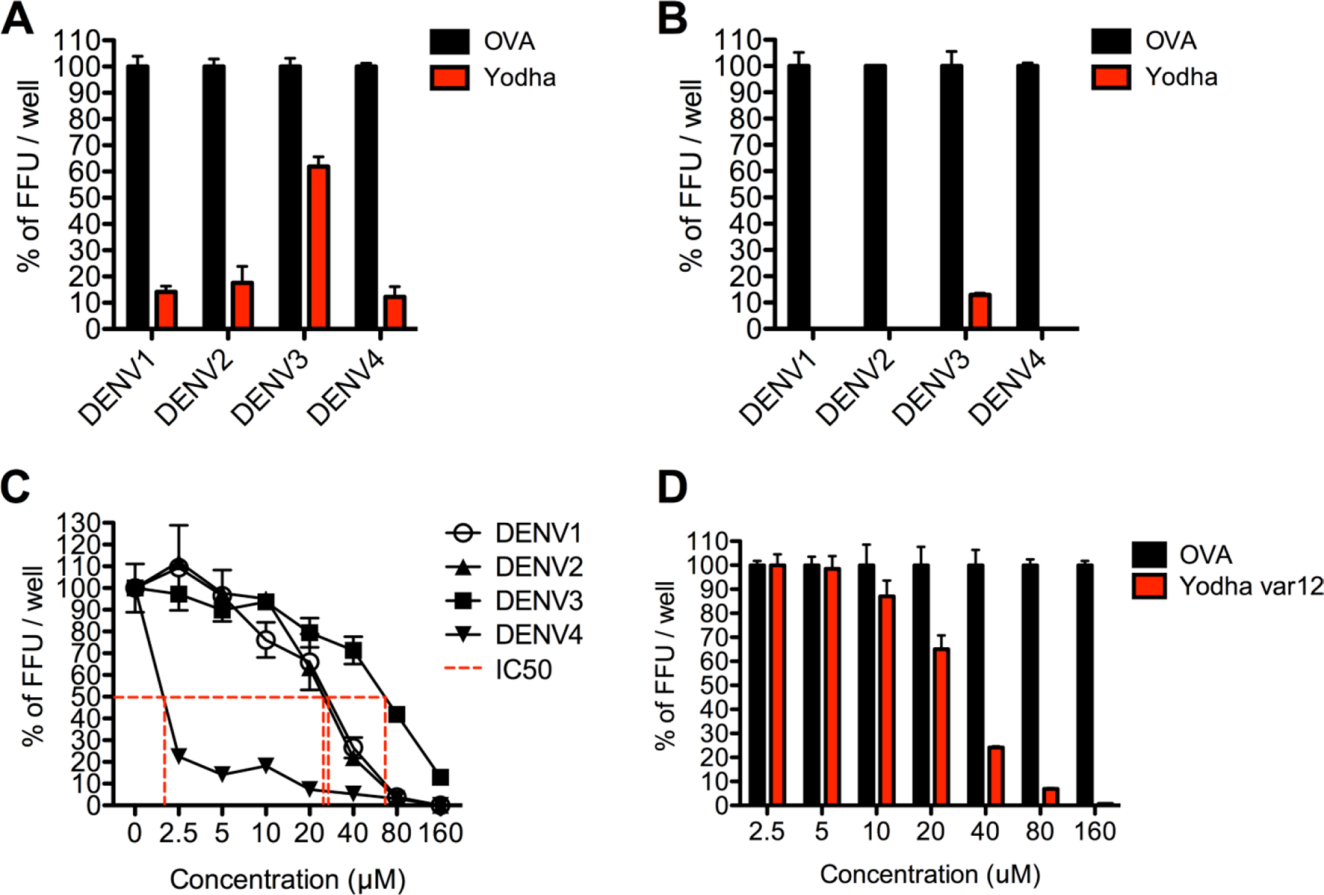
Yodha inhibits all four strains of DENV. Dengue virus strains 1, 2, 3 and 4 were treated with wither Yodha or control OVA peptide at (**A**) 50 µM (**B**) 160 µM concentration and the % of viral focus forming units were determined. (**C**) IC_50_ measurement of Yodha in DENVs infection by FFA. (**D**) Dose escalation of truncated Yodha variant 12 against DENV3 infection shows that the truncated version is more efficient than the full length Yodha peptide. OVA peptide was used a negative control.

ZIKV is closely related to dengue virus (DENV), and next, we tested whether Yodha was effective against all four serotypes of DENV. Yodha peptide efficiently inhibited DENV1, DENV2, and DENV4, but not DENV3 strains at 50 µM concentration (Fig. 7A). However, when the concentration of Yodha was increased to 160 µM, it was very efficient against DENV3 (Fig. 7B). The half-maximal inhibitory concentration (IC_50_) of the Yodha peptide was 25 µM, 2 µM, 70 µM and 30 µM for DENV1, 2, 3, and 4, respectively. Since the IC_50_ of Yodha against DENV3 was higher than other strains, we tried the truncated Yodha variant 12, which was significantly more effective against ZIKV (Fig. 5D). The truncated Yodha variant 12 was very efficient against DENV3 (Fig. 7D) as compared to the full length Yodha peptide. The IC_50_ of Yodha variant 12 for DENV3 was 30 µM while that for full-length Yodha peptide was 70 µM. Taken together, our data suggest that Yodha peptide is effective against not just ZIKV but DENV as well.

## Discussion

Host defense peptides, which constitute the ancient arm of the innate immune system, confers protection to the host. The amphibian host defense peptide Yodha acts against Zika viruses and could be developed as a promising antiviral for the following reasons. First, it acts on the virus directly and causes lysis of the virus. Second, Yodha acts on all lineages of ZIKV. We independently sequenced each of the four ZIKV strains and found that P6-1966, MR-1947, and Dak-194 differed at the amino acid level from PR-2015 by 1.1%, 3.2%, and 3.0%, respectively^31^. Also, MR-1947 diverged from PR-2015, more notably in the structural (4.4%) than non-structural proteins (2.9%). Nonetheless, Yodha peptide targets all four different lineages, suggesting that it may be targeting a motif that is conserved among all ZIKV. Third, Yodha peptide is nontoxic to human RBC; the IC_50_ of this peptide is 20 µM (0.052 mg/ml) and nontoxic at 1000 µM (2.6 mg/ml), giving a broad range of doses that can be administered. Fourth, Yodha peptide rapidly neutralizes the virus, at least within 5 min of exposure to the virus, which is an advantageous feature in a therapeutic^32^. Fifth, we showed that both L- and D-enantiomeric forms of Yodha peptides work against ZIKV (Fig. 2D), and this is significant because unlike the naturally occurring L-form, the D-form is more stable in vivo as is less susceptible to endogenous proteases^33^.

It is currently unclear as to which region of the ZIKV particle is targeted by the Yodha peptide. Since it is effective against ZIKV viruses that circulated over a span of 68 years (1947 – 2015), likely, the peptide targets a conserved motif within the envelope, and possibly, the matrix protein conserved among all of these viruses. Our EM studies suggest that the Yodha peptide may induce viral lysis. Hydrophobicity has been known to be the main driving force for the integration of transmembrane segments into the lipid bilayer of the protein^34–36^. Interestingly the Yodha peptide as well as the naturally occurring variant peptides shown in Fig. 7 have a common hydrophobic N terminus (Fig. 1C) to which the activity of the peptide could be mapped. This suggests that the peptide destabilizes a virus particle by integrating into the viral lipid bilayer using the hydrophobic N-terminus.

It is indeed remarkable that naturally occurring variants of the Yodha peptide was also active against ZIKV. These peptides came from frogs with natural habitats that span the globe – India, China, Sri Lanka, Myanmar, USA, Canada, Mexico, Korea, Russia, and Europe. It is highly unlikely that these amphibians produce these peptides to combat ZIKV, but instead, these peptides confer survival advantage against some common amphibian pathogen. These peptides might act via “pattern recognition” analogous to pattern recognition receptors and that ZIKV coincidentally shares conserved patterns/motifs.

Our findings provide the proof-of-principle that the Yodha peptide could be developed as a useful antiviral compound to ZIKV and potentially DENV. The direct virucidal activity of this peptide on ZIKV, as well as its low toxicity on human RBCs, make it a promising candidate for development to combat ZIKV and DENV. The pharmaceutical industry could build on our findings and optimize and develop robust delivery systems to produce successful anti-ZIKV and anti-DENV treatments.

## Materials and methods

All methods were carried out in accordance with approved guidelines. All experiments were performed in accordance with relevant guidelines and regulations. All procedures on amphibians and mice were carried out with the approval of the Ethical Committee at the Rajiv Gandhi Center for Biotechnology in India and IACUC at Emory University, respectively. This study was carried out in compliance with the ARRIVE guidelines.

### Cells and viruses

Vero cells were cultured in Dulbecco’s modified Eagle’s medium (DMEM; Lonza) supplemented with 10% FBS (FBS; Atlanta Biologicals), 1% Pen/Strep, and 1% HEPES. We used ZIKV strains (PRVABC59, MR-766, DakAr, and P6-740)^37^. ZIKV infection experiments were conducted under biosafety level 2 + (BSL2 +).

### Peptides

Briefly, mRNAs were initially isolated from frogs in the Western Ghats regions of S. India by stimulus-driven secretion, converted to cDNA, amplified by PCR, and sequenced as described^38^. The amphibian sequences were then used to synthesize peptides, and this was done at Genemed Synthesis Inc. (San Francisco, CA), Neo Scientific, and Dr. Brian Evavold's laboratory at Emory University. As a control, OVA_257-264_ peptide (Invivogen) was used.

### Focus forming assay

Peptides were incubated with ZIKV (100 FFU/well) for 2 h at 37 °C. This incubated mixture was used to infect Vero cells for 1.5 h at 37 °C. Cells and inoculum were overlaid with 2% methylcellulose solution (OptiMEM; Gibco) and incubated for 72 h at 37 °C. Cells were washed with PBS and fixed with a 1:1 methanol/acetone mixture for 30 min. Cells were blocked with 5% milk/PSB at room temperature for 20 min and incubated with primary antibody (anti-flavivirus mouse 4G2^10^ antibody) for 2 h at 37 °C. Then, cells were incubated with secondary antibody (HRP-conjugated goat anti-mouse IgG, Cell Signaling) for 1 h at 37 °C. Cells were developed with TrueBlue Peroxidase Substrate (KPL). Plates were read on a CTL-ImmunoSpot S6 Micro analyzer.

### Hemolysis toxicity assay

Single donor human red blood cells (Innovative Research) were washed in PBS (pH 7.4) with three times centrifugation at 500 × g for 5 min. Serially diluted frog peptides or OVA control peptide were prepared in V-bottom 96 well plates and mixed with washed 2 × 10^7^ human RBC per each well for 1 h at 37 °C. PBS solution was used as a negative control (0% lysis), and 0.1% Triton X-100 in PBS was used as 100% lysis. The plates were centrifuged at 300 × g at 4 °C for 5 min to pellet the intact RBCs and supernatant of each well was measured by absorbance at 450 nm.

### LDH assay

Cytotoxicity Detection Kit was purchased from Pierce LDH cytotoxicity assay kit, Thermo Scientific. The LDH assay was performed according to the manufacturer's instructions. Briefly, 1 or 1.5 × 10^4^ cells / well were plated in a 96-well plate and treated with Yodha peptide at various concentrations as indicated at 37 °C for 5 h. Absorbance at 490 nm and 680 nm is measured using a plate-reading spectrophotometer (Synergy 2 multi-mode reader, Biotek) to determine LDH activity.

### Immunofluorescence

Vero cells were grown and infected with ZIKV (PRVABC59) at an MOI of 1 on glass coverslips for 30 min and washed three times with phosphate-buffered saline (PBS) prior to fixation. Cells were then fixed with 4% paraformaldehyde solution for 10 min and permeabilized in 0.2% Triton X-100 for 12 min at room temperature. Cells were blocked in protein Block Solution Serum-Free (Dako) for 1 h and stained with primary (mouse 4G2 monoclonal antibody and rabbit anti-alpha tubulin polyclonal antibody, Millipore) and secondary (donkey anti-mouse Alexa-488 and Alexa-594, Thermo Fisher). After wash, samples were mounted with antifade mountant with DAPI (Thermo Fisher). Images were taken with an Olympus Fluoview FV1000 microscope using FV10-ASW2.1 acquisition software (https://www.olympus-lifescience.com/en/support/downloads/fv10i_vw_license/).

### qRT-PCR

Total RNA was extracted from mock- or ZIKV- infected Vero using the RNeasy Plus mini kit (Qiagen). For qRT-PCR, total RNA was converted to complementary DNA using the High-Capacity cDNA Reverse Transcription Kit (Applied Biosystems) using random hexamers. For quantification of viral RNA, qRT-PCR was performed using TaqMan Gene Expression Master Mix (Applied Biosystems) by the manufacturer’s instructions. Primers used for RT-PRC were described previously by Quicke et al.^37^ Viral RNA was normalized to cellular GAPDH and relative to mock infection controls.

### ZIKV negative staining for EM

ZIKV samples were fixed with 4% buffered paraformaldehyde before negative staining. 5 µl of Zika sample was then deposited onto a 400-mesh carbon-coated copper grid that had been treated by glow-discharged for 20 s and allowed 5 min for the sample to settle in a covered glass dish. The grids with samples were then quickly washed by touching the sample side on two drops double distilled water, wick with filter paper, and then stained with 1% phosphotungstic acid (PTA) for 15 s before removing PTA with filter paper. Zika virus was imaged on a JEOL JEM-1400 transmission electron microscope (JEOL Ltd., Tokyo, Japan) equipped with a Gatan US1000 CCD camera (Gatan, Pleasanton, CA).

### Mouse Zika virus infection

All mouse studies were approved by the IACUC of Emory University. C57BL/6 mice were purchased from The Jackson Laboratory. All mice were maintained in specified pathogen-free in accordance with the institutional guidelines of Emory University’s Animal Care and Use Committee. All control and experimental mice were age- and sex-matched. For viral titer assessment, Wild-type C57BL/6 J mice were injected with 2 mg of an anti-Ifnar1 blocking antibody (MAR1-5A3) by the intraperitoneal route 24hrs prior to infection^11^. Mice were anesthetized with isoflurane and inoculated by the subcutaneous route into both rear footpads with 5 × 10^4^ PFU of ZIKV PRVABC59 (1 × 10^5^ PFU total inoculum per mouse) in a 20µL inoculum diluted in Hanks balanced salt solution (HBSS) supplemented with 1% heat-inactivated FBS. For analysis of morbidity by ZIKV viral infection, we infected 5- to 6-week-old IFNAR KO mice with ZIKV MR766 (100 PFU total inoculum per mouse). Mice were monitored daily for morbidity and mortality.

### Measurement of tissue viral burden

At 3 days post-infection (dpi), blood was collected and allowed to clot in serum separator microtainer tubes (BD Biosciences). Viral RNA was isolated from serum (viral RNA isolation kit; Zymo research) and quantitated using one-step qRT-PCR (SuperScript III Platinum One-Step qRT-PCR kit; Invitrogen) with a previously described primer and probe set targeting the prM/E region of the ZIKV PRVABC59 genome^31^. At 6dpi, eyes and spleens were harvested into QiaZol (Qiagen) and homogenized with ceramic beads (Omni Bead Ruptor). Total RNA was isolated (RNeasy Plus Universal mini kit, Qiagen), and viral RNA was quantitated using two-step qRT-PCR as previously described^31^. For the serum samples, RNA standards were used to calculate ZIKV copies/mL within each sample. For the tissue samples, relative ZIKV RNA quantities were calculated as the fold-increase over naïve controls after normalization to the amount of GAPDH within each respective sample. All qRT-PCR data is represented as a relative percentage as compared to the average of the OVA controls, calculated as percent ZIKV RNA relative to OVA = ([Yodha_ZIKV RNA_] / [Average OVA_ZIKV RNA_]) * 100.

### Statistical analysis

Statistical significance was verified by the Student's t-test, two-way ANOVA.
